# Correlation Between Dental Age, Chronological Age, and Cervical Vertebral Maturation in Patients with Class II Malocclusion: A Retrospective Study in a Romanian Population Group

**DOI:** 10.3390/children12040398

**Published:** 2025-03-21

**Authors:** Mircea Ghergie, Cristina Dora Ciobotaru, Ruxandra Pop, Ioana Colceriu-Șimon, Olimpia Bunta, Mihaela Pastrav, Dana Feștilă

**Affiliations:** Department of Orthodontics, Faculty of Dentistry, “Iuliu Hațieganu” University of Medicine and Pharmacy, 400012 Cluj-Napoca, Romania; mircea.ghergie@umfcluj.ro (M.G.); ruxandra.pop@elearn.umfcluj.ro (R.P.); simon.ioana@umfcluj.ro (I.C.-Ș.); nemes.olimpia@umfcluj.ro (O.B.); mihaela.pastrav@umfcluj.ro (M.P.); dana.festila@umfcluj.ro (D.F.)

**Keywords:** chronological age, dental age, skeletal age, cervical vertebral maturation, growth peak, orthodontic treatment, class II malocclusion

## Abstract

**Background/Objectives**: The relationship between chronological age, dental age, and cervical vertebral maturation is critical for assessing the reliability of dental age as an indicator of skeletal age and for identifying the patient’s growth peak. This assessment facilitates the planning of appropriate orthodontic-orthopedic treatment. **Methods**: This retrospective observational study analyzed data from the Clinical Department of Orthodontics and Dento-Facial Orthopedics in Cluj-Napoca, Romania. The sample included 73 patients with Class II malocclusion (31 males and 42 females), with data obtained from orthopantomography and lateral cephalometric radiographs. Dental age was evaluated using both the Demirjian method and the Chronology of Eruption method. Skeletal age was determined based on Baccetti’s cervical vertebral maturation (CVM) staging method. **Results**: A strong and statistically significant correlation was found between cervical vertebral maturation and chronological age (r = 0.81, *p* < 0.001), as well as between cervical vertebral maturation and dental age assessed using the Demirjian method (rs = 0.72, *p* < 0.001). Additionally, a significant correlation was observed between cervical vertebral maturation and dental age assessed using the Chronology of Eruption method (rs = 0.78, *p* < 0.001). **Conclusions**: The correlation found between dental age and skeletal maturity suggests that dental age (DA) assessment might serve as a supplementary tool for estimating a patient’s growth peak in Class II malocclusion cases. Future research exploring the use of artificial intelligence (AI) in panoramic radiograph analysis could improve the accuracy and consistency of DA assessment, contributing to more reliable skeletal maturity evaluations.

## 1. Introduction

The chronological age (CA) influences the process of growth and maturation of the human body [1,2]; however, individuals of the same CA can exhibit significant differences in developmental stages [3,4,5]. This discrepancy highlights the distinction between CA and biological age [6], the latter being determined by specific biological parameters that indicate a patient’s developmental stage [7,8].

While the CA of an individual can be easily determined by the date of birth [9], biological age has historically been assessed through different indicators, including weight and height measurements, secondary sexual characteristic development, and, more recently [10,11], skeletal age (SA) estimation via radiographic analysis [12,13]. Among these, SA assessment is considered the most reliable for determining a patient’s developmental stage [14,15]. The optimal method for assessing SA has been a longstanding subject of debate among specialists [16,17]. The most commonly used radiographs for bone maturity evaluation include hand–wrist radiographs and lateral cephalometric radiographs (Lceph) [18,19,20]. While the hand–wrist radiograph has been traditionally considered the gold standard for assessing bone maturity as an indicator of biological age [21], its major drawback is the additional radiation exposure to the patient [18,22]. Since cephalometric radiography is routinely required at the beginning of orthodontic treatment for diagnostic measurements and treatment planning [23,24], it represents a practical alternative for evaluating the patient’s developmental stage without subjecting the patient to further radiation exposure through hand–wrist radiography [25,26].

Consequently, lateral cephalometric radiography (Lceph) is recognized as a reliable method for assessing bone maturity [27,28], offering the advantage of simultaneously serving both diagnostic and treatment planning purposes [22,29].

Assessing bone maturity is crucial for estimating the timing of the pubertal growth peak [30,31]. The clinical significance of growth assessment extends beyond orthodontics, as skeletal maturity plays a fundamental role in various fields of dentistry, including orthognathic surgery, implantology, and prosthodontics [32,33]. Accordingly, the clinical applicability of assessing growth variations during adolescence and preadolescence lies in therapeutic planning associated with the pubertal growth spurt [34], particularly for guiding mandibular growth and achieving predictable, stable therapeutic outcomes [35,36].

Class II malocclusion, the second most prevalent orthodontic disorder after Class I malocclusion, affects approximately 20–25% of the global population [37]. This malocclusion is characterized by various dental and alveolar traits, primarily involving mandibular retrusion [38,39]. Orthodontic treatment strategies often aim to promote mandibular growth, with orthopedic advancement of the mandible being a key treatment objective [40]. Mandibular advancement can be achieved using functional appliances, whether removable or fixed [41]. However, treatment effectiveness and patient compliance depend significantly on precise timing, as modifications in mandibular growth should coincide with the patient’s growth peak [42,43]. Despite its advantages [44], performing Lceph at an early age to capture the growth peak can be challenging due to potential positioning errors and reduced image quality [45], as younger patients may struggle to maintain stillness during radiographic procedures [46]. Additionally, Lceph should not be overused due to radiation exposure, particularly given that most orthodontic patients are children [47].

Given the significance of accurately determining the growth peak, especially in Class II malocclusions, research into alternative skeletal maturity indicators is warranted [43]. In the Romanian population, the prevalence of Class II malocclusion aligns with global trends [48], emphasizing the importance of optimizing treatment timing within this demographic.

This study investigates the correlation between chronological age (CA), dental age (DA), and cervical vertebral maturation (CVM) in Class II patients from a Romanian population. Specifically, we seek to determine the accuracy of DA as an alternative predictor of skeletal maturity, thereby reducing the need for repeated Lceph radiographs in orthodontic diagnostics. Conversely, if no significant correlation is found, this would indicate that DA cannot be reliably used as an alternative to CVM assessment.

DA was assessed using two methods—the Demirjian method (Dmet) and the Chronology of Eruption method (CEMet)—via orthopantomography (OPG), while CVM was evaluated using Lceph radiographs based on Bacetti’s method [49]. The collected data were then correlated with the CA of the patients.

The results of this study provide insights into the potential of DA as a tool for approximating CVM through clinical examination alone, thus minimizing the need for repeated Lceph exposures. While Lceph remains essential for treatment planning, its use should ideally be limited to the initial phase of orthodontic therapy and follow-up assessments only when necessary.

## 2. Materials and Methods

This study is a retrospective observational analysis based on data collected from the archive of the Clinical Department of Orthodontics and Dento-Facial Orthopedics, Cluj-Napoca, Romania, between May 2020 and June 2024. After applying the inclusion and exclusion criteria, 73 patients diagnosed with Class II malocclusion (31 males and 42 females) were selected from the entire database. The final sample (*n* = 73), aged between 6 and 15 years (mean age 12.03 ± 2.2 years), was evaluated using cephalometric files and panoramic radiographs (OPG).

To ensure consistency and accuracy in data evaluation, three independent observers assessed the panoramic (OPG) and lateral cephalometric (Lceph) radiographs. Any minor variations in interpretation were resolved through standardized guidelines and consensus discussions, ensuring a uniform approach to skeletal and dental age assessments.

Participation in this study was based on informed consent obtained from the patients’ parents or legal guardians, in accordance with ethical guidelines given by the Ethics Committee of U.M.F. Cluj-Napoca no. 20/20.01.2014, and on adherence to the following inclusion and exclusion criteria:

(i) Inclusion criteria—patients diagnosed with Class II malocclusion, age range: 6–15 years, normal growth and development, without systemic conditions affecting bone development (as determined through medical history and anamnesis), availability of clear and accurate panoramic radiographs and lateral cephalometric (Lceph) images, the availability of clear and accurate panoramic radiographs and Lceph images, first-time orthodontic patients with no prior history of orthodontic treatment.
(ii) Exclusion criteria—patients younger than 6 years or older than 16 years, multiple dental aplasia, severe metabolic disorders, incomplete records in the database.

Chronological age (CA) was determined by calculating the time from the patient’s birth date to the date of the panoramic radiograph or by extracting the information from the patient’s medical record.

Dental age (DA) was assessed using two methods: the Demirjian method (DMet)—evaluated based on crown and root dental development stages observed in OPG—and the Chronology of Eruption method (CEMet)—determined by analyzing the development of permanent teeth in relation to the average eruption age in Romanian children.

The lateral cephalometric radiographs (Lceph) were evaluated to establish the cervical vertebral maturation stage (CVM) using Baccetti’s method [18].

### 2.1. Dental Age Assessment

#### 2.1.1. Demirjian Method

**Figure 1:**
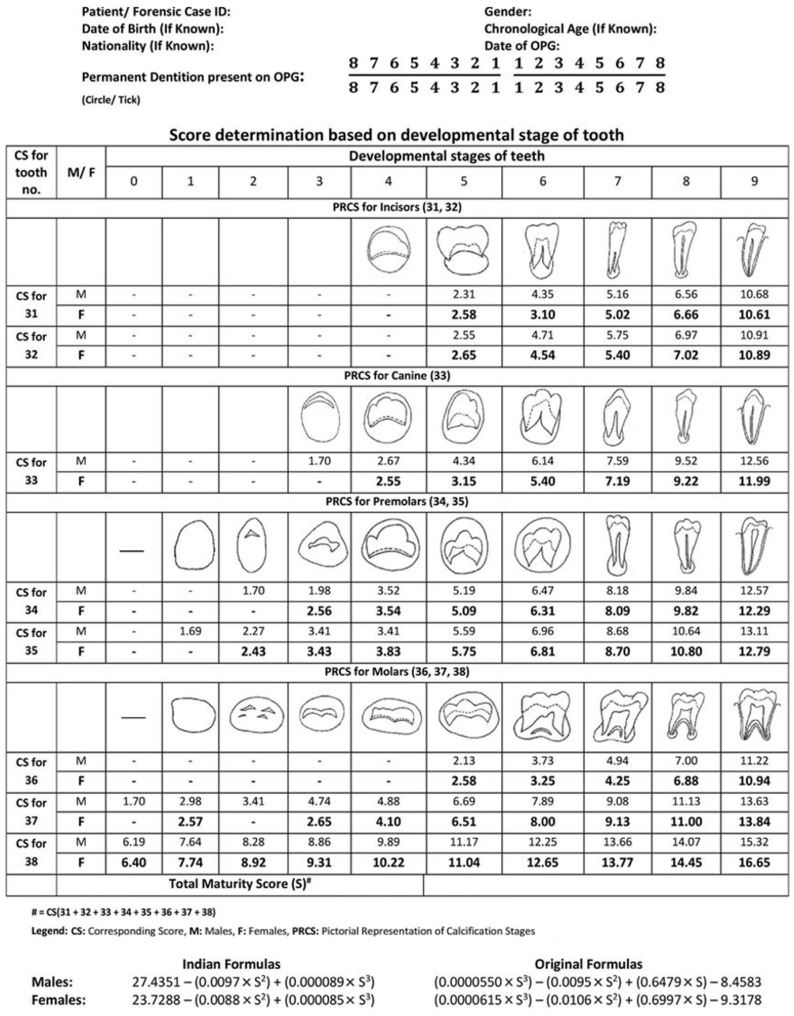
Determination of score based on developmental stages of the tooth [51].

DA was primarily determined using the Demirjian method (DMet), which evaluates the crown and root development stages of teeth on OPGs [50]. Each patient was assigned a score based on Demirjian’s developmental chart (Figure 1).

The assessment focused on the left mandibular quadrant (from incisors to second molar). If a tooth was missing in this quadrant, the right homologous tooth was evaluated.

The developmental stages in the Demirjian method are as follows:

A: Mineralized cusp tips not coalesced;

B: Coalesced cusp tips;

C: Crown formation halfway completed;

D: Crown fully formed;

E: Root initiation;

F: Root length at least equal to the crown;

G: Root walls parallel with open apices;

H: Apices closed.

**Figure 2:**
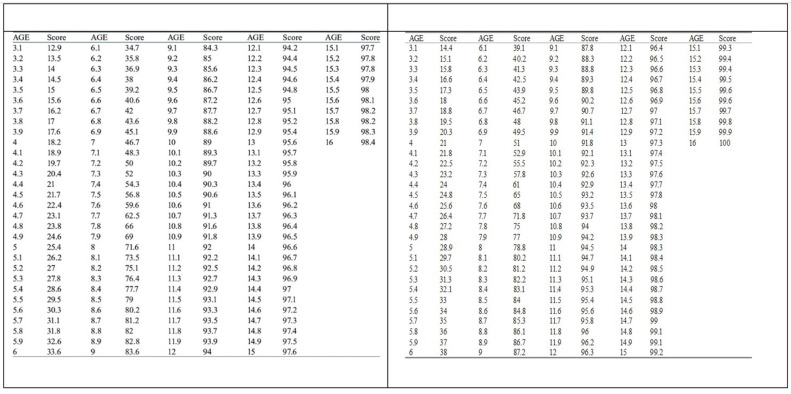
Conversion tables (male and female) for establishing DA according by the DMet [52].

The DA was derived by converting the developmental score using a standardized conversion table based on patient sex (Figure 2).

To ensure accuracy, panoramic radiographs were initially assessed and scored by a primary evaluator and subsequently verified by a second observer. Digital radiographs allowed magnification for detailed dental morphology assessment.

#### 2.1.2. Chronology of Eruption Method (CEMet)

**Figure 3:**
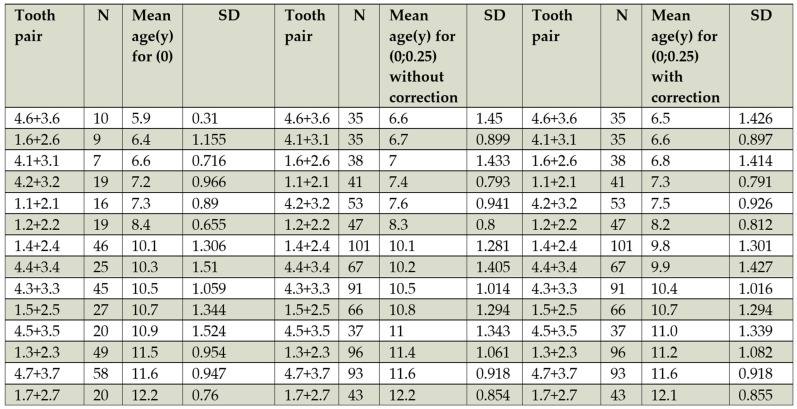
The mean age and the sequence of eruption of permanent teeth [53].

As a supplementary approach, DA was also assessed using the Chronology of Eruption method (CEMet). This method, though less precise than DMet, estimates DA based on the development of the last permanent tooth with at least half of its root formed, relative to the mean eruption age in Romanian children (Table 1, Figure 3) [53].

### 2.2. Skeletal Maturity Assessment: Cervical Vertebral Maturation (CVM) Method

**Figure 4:**
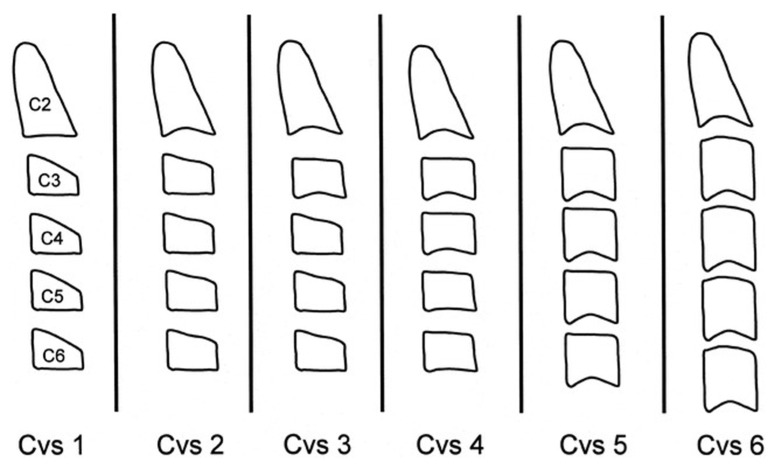
Stages of CVM [49].

Lceph images were analyzed to determine CVM, an indicator of skeletal maturity, based on the morphology of the first four cervical vertebrae. The CVM assessment followed Baccetti’s [49] method, which classifies skeletal maturity into six cervical developmental stages (CVM1–CVM6) based on the shape of the vertebral bodies and the presence of concavities on their lower borders (Figure 4), in order to anticipate the occurrence of the peak in mandibular growth, which is assumed to happen between CVM3 and CVM4. In the CVM1 stage, the lower borders of C2, C3, and C4 are flat, while C3 and C4 exhibit a trapezoidal shape, indicating that the growth peak is expected to occur in more than two years. In CVM2, a concavity appears on the lower border of C2, while C3 and C4 remain unchanged, suggesting that the growth peak is anticipated within approximately one year. As the skeletal maturity progresses to CVM3, concavities develop on the lower borders of both C2 and C3, and C3 and C4 transition into a trapezoidal or rectangular horizontal shape, signifying that the growth peak is imminent. In CVM4, concavities are present on the lower borders of C2, C3, and C4, with C3 and C4 taking on a rectangular horizontal shape, indicating that the growth peak has already occurred one to two years prior. At CVM5, at least one of C3 or C4 adopts a square shape while the concavities on all three vertebrae persist, suggesting that at least one year has passed since the peak of mandibular growth. Finally, in CVM6, one of C3 or C4 assumes a rectangular vertical shape, while concavities remain present, marking the end of the growth peak more than two years earlier [54].

### 2.3. Statistical Analysis

Statistical analysis was performed using SPSS Statistics v29.0.1 (IBM Corp., Armonk, NY, USA). Continuous variables were reported as mean ± standard deviation (SD), and categorical variables were reported as frequencies. Normality of continuous variables was assessed using Shapiro–Wilk’s test, and equality of variances was tested using Levene’s test. Correlation analysis was performed using point-biserial correlation coefficient (rpb). A two-tailed *p* < 0.05 was considered statistically significant.

## 3. Results and Discussion

This section was organized into subheadings, offering a concise and precise presentation of the experimental results, their interpretation, and the conclusions derived from the findings.

The mean chronological age (CA) of the study population was 12.03 ± 2.2 years, while the mean dental age (DA) was 13.28 ± 2.36 years using the Demirjian method (DMet) and 12.07 ± 2.48 years based on the Chronology of Eruption method (CEMet). The most prevalent cervical vertebral maturation (CVM) stage was CVM 4, observed in 25 patients, followed by CVM 3 with 15 patients. CVM 2 and CVM 1 were found in 12 and 10 patients, respectively. The lowest frequencies were recorded in CVM 5 (6.85%) and CVM 6 (8.22%). The distribution of these variables is presented in Table 1(a,b).

Among the 73 enrolled patients, 31 were male and 42 were female. In CVM 1, the male-to-female ratio was equal (6.85% each). Female participants predominated in CVM stages 2, 4, 5, and 6, with the largest discrepancy observed in CVM 4 (21.92% females vs. 12.33% males). Conversely, CVM 3 was the only stage where males outnumbered females (eight vs. seven patients). Statistical analysis revealed no significant association between CVM stage and sex (*p* = 0.90), as well as no correlations between CA and sex (*p* = 0.58), DA (DMet) and sex (*p* = 0.66), or DA (CEMet) and sex.

**Figure 5:**
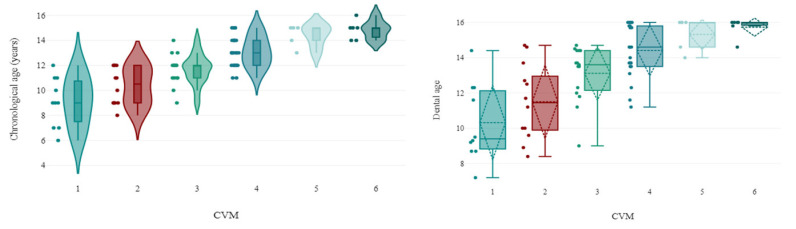
CVM distribution based on chronological age (CA) and dental maturation (DMet).

The distribution of CVM stages relative to CA is illustrated in Figure 5. CVM 1 was observed in children aged 6 to 12 years, with a peak incidence at 9 years. CVM 2 had a mean CA of 10.33 ± 1.5 years, while CVM 3 was associated with a mean CA of 11.67 ± 1.23 years. The majority of CVM 4 cases occurred between ages 11 and 15, peaking at 13 years. The mean CA for CVM 5 and CVM 6 was 14.4 ± 0.89 years and 14.83 ± 0.75 years, respectively.

The CVM distribution by DA (DMet) is represented in Figure 3. The DA range for CVM 1 was 7 to 14 years, with a mean of 10.32 ± 2.21 years. The mean DA for CVM 2 was 11.5 ± 2.17 years, and for CVM 3, it was 13.11 ± 1.58 years. CVM 4 patients had a DA range of 11 to 16 years, with a mean of 14.41 ± 1.46 years. The mean DA for CVM 5 and CVM 6 was 15.32 ± 0.95 years and 15.73 ± 0.56 years, respectively.

The mean DA assessed by the CEMet differs from the one assessed by the DMet, being reduced by about one year for each stage of CVM, as follows: 9.2 ± 2.3 years for the CVM1 stage; 10.25 ± 1.6, 11.4 ± 1.4, and 13.2 ± 1.73 years for the CVM 2, 3, 4 stages; and 14.6 ± 1.14 and 15.33 ± 1.03 years for the CVM stages 5 and 6. Table 2 summarizes the mean DA obtained using both methods.

**Figure 6:**
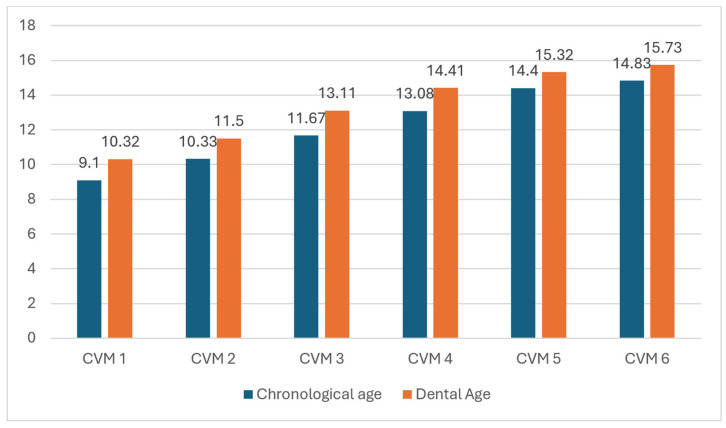
CVM frequency by CA and DA.

Figure 6 highlights the discrepancies between CA and DA across CVM stages. The largest differences were found in CVM 3 and CVM 4, with mean deviations of 1.44 ± 0.517 years and 1.33 ± 0.38 years, respectively. The smallest discrepancies were observed in CVM 5 (0.92 ± 0.58 years) and CVM 6 (0.9 ± 0.43 years). In CVM 1 and CVM 2, the mean differences were 1.22 ± 0.93 years and 1.17 ± 0.76 years, respectively. At all CVM stages, DA was consistently higher than CA (Table 3).

**Figure 7:**
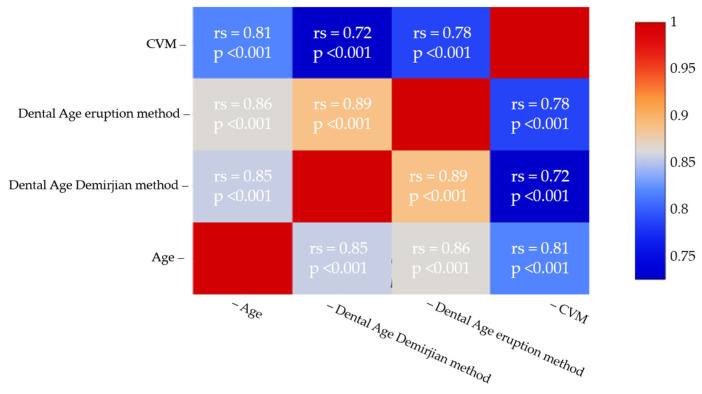
Spearman test results for the correlation between CVM—CA and CV—MDA.

Spearman’s correlation test demonstrated a strong positive correlation between CVM and CA (rs = 0.81, *p* < 0.001). Significant correlations were also observed between CVM and DA (DMet: rs = 0.72, *p* < 0.001; CEMet: rs = 0.78, *p* < 0.001) (Figure 7).

### 3.1. Mean Chronological Age by CVM Stage and Gender

A review of six relevant studies [20,29,30,55,56,57] consistently demonstrated a relationship between DA and CVM, evaluated using the Demirjian method and Baccetti’s CVM method. According to Baccetti, the peak growth spurt occurs between CVM stages 3 and 4. In the present study, the mean CA at CVM 3 was 11.42 years in females and 11.87 years in males, confirming that females reach skeletal maturity earlier. Table 4 provides a detailed summary.

Findings from Magat et al. [30] support this observation, reporting a mean CA of 12.42 ± 0.85 years for females and 13.15 ± 1.46 years for males in CVM 3. The study also noted that the mean CA was lower for females at each CVM stage. These results are consistent with the findings in this paper, except for CVM 6, in which the mean CA for girls was higher. A retrospective study on the French population [29] found similar results for CVM 3, with a mean CA of 11.21 ± 1.32 for females and 12.20 ± 1.70 for males. Similar results were also reported by a study on the Iranian population published by Mollabashi et al. [55] (11.97± 1.38 for males and 11.59± 1.40 for females).

Regarding the mean CA for CVM 3, all the studies, including our work, reported similar results; however, the difference between the mean CA of both females and males decreased as CVM progressed. These findings indicate that while females mature faster initially, the difference in CA between sexes diminishes as CVM progresses.

### 3.2. Correlation Between CVM and CA and DA

To our knowledge, all selected studies [20,29,30,55,56,57] have investigated the correlation between CVM and DA. The present study further analyzed the relationships between CVM, CA, and DA.

Regarding the correlation between CVM and DA and CVM and CA, Brotons et al. [29] evaluated a number of 192 patients. Using Spearman’s correlation test, they sought to determine whether correlations exist between CVM, DA, and CA, similar to the approach taken in the present study. They found moderate results, with a weaker correlation between the three variables than what we have determined in this work. A strong correlation between CA and DA was reported in both genders (0.79–0.8), while a moderate correlation was determined between CVM and DA (0.3–0.4).

A similar study conducted on the eastern Romanian population by Savin et al. [57], analyzed a sample of 88 patients, investigating the association between CVM and the developmental stages of individual teeth. Unlike our study, which assessed DA as a whole, their approach aimed to correlate CVM with the maturation of specific teeth. Their results showed moderate to low correlations for most teeth, except for the central and lateral incisors, which exhibited weaker associations. The strongest correlation was found between CVM and the mandibular canine, leading the authors to suggest that the mandibular canine is the most reliable dental indicator of skeletal maturity. These findings align with a recent meta-analysis of 77 studies, which also identified the mandibular canine, first and second premolars, and first molar as the teeth most strongly correlated with CVM. However, due to the heterogeneity of the data, these results should be interpreted with caution [58].

Further supporting these findings, Valizadeh et al. and Pooja et al. [20,56] found a moderate correlation between CVM and DA, as follows: Valizadeh et al. found the strongest correlation between DA of the second molar, first molar, and lateral incisor and CVM, while Pooja et al. [20] found the highest correlation between DA of the mandibular canine and second molar and CVM. These results are consistent with those reported by Savin et al. [55], conducted on a Romanian sample group in 2019. Similarly, Magat et al. [30] reported a moderate correlation between CVM and DA (0.636 for females and 0.695 for males).

As seen across these studies, correlations between CVM and DA range from strong [20,30,55,56,57], to moderate [29,56] and weak [29]. In contrast, the present study found very strong correlations between CVM and DA, CVM and CA, and CA and DA. The strongest correlation was observed between DA determined by the Demirjian method (DMet) and DA determined by the Chronology of Eruption method (CEMet). This suggests that CEMet could be a valuable tool for estimating DA and, consequently, CVM. However, it is important to note that CEMet relies on eruption tables specific to the Romanian population, and its accuracy is population-dependent. The study by Savin et al. [57] on an Eastern Romanian population can be seen as complementary to our research, which was conducted on a Transylvanian population.

One limitation of the present study is that only patients with Class II malocclusion were included, which means that the findings may not be applicable to other types of malocclusions. However, this specific malocclusion was chosen due to the importance of timing treatment during the growth spurt. In Class II malocclusion cases, early detection of the growth peak is critical for guiding mandibular advancement. Once the pubertal growth spurt has passed, treatment options become more limited, often requiring camouflage strategies or, in severe cases, a combination of orthodontics and orthognathic surgery [59,60]. Given this clinical significance, understanding the potential role of DA in predicting skeletal maturity within this malocclusion is particularly relevant. Additionally, the sample size (*n* = 73) was relatively small, and DA was assessed as an overall measure rather than for individual teeth. Future research should expand on this by investigating correlations between CVM and the development of specific teeth to refine DA-based skeletal maturity assessments.

## 4. Conclusions

The patient’s growth spurt might be assessed by monitoring cervical vertebral maturation (CVM). This study demonstrated a correlation between CVM, dental age (DA), and chronological age (CA) in patients with Class II malocclusion. Therefore, CA and DA might be indicators for estimating CVM and predicting the growth spurt, potentially minimizing additional radiation exposure by avoiding repeated lateral cephalometric radiographs.

Given the clinical significance of Class II malocclusion, accurately determining the growth peak is essential for guiding mandibular advancement treatment. Future research exploring the use of artificial intelligence (AI) in panoramic radiograph analysis could improve the accuracy and consistency of DA assessment, contributing to more reliable skeletal maturity evaluations.

## Figures and Tables

**Table 1:** (**a**) Age distribution according to CA * and DA ** (Dmet ^1^ and CEMet ^2^). (**b**) Distribution of CVM stages.

(**a**)	
**Variable**	**Mean ± SD**
Age (years)	12.03 ± 2.2
DA DMet ^1^ (years)	13.28 ± 2.36
DA CEMet ^2^ (years)	12.07 ± 2.48
(**b**)	
**CVM ^3^ Stage**	**Frequency**
CVM 1	10 (13.7%)
CVM 2	12 (16.44%)
CVM 3	15 (20.55%)
CVM 4	25 (34.25%)
CVM 5	5 (6.85%)
CVM 6	6 (8.22%)

^1^ Dental age Demirjian method; ^2^ dental age Chronology of Eruption method; ^3^ Cervical vertebrae maturation; * chronological age; ** dental age.

**Table 2:** Frequency of patients in each CVM stage according to patient gender and CA and DA.

Variable	CVM 1 (*n* = 10)	CVM 2 (*n* = 12)	CVM 3 (*n* = 15)	CVM 4 (*n* = 25)	CVM 5 (*n* = 5)	CVM 6 (*n* = 6)
Age (years)	9.1 ± 1.97	10.33 ± 1.5	11.67 ± 1.23	13.08 ± 1.22	14.4 ± 0.89	14.83 ± 0.75
Male (gender)	5 (6.85%)	5 (6.85%)	8 (10.96%)	9 (12.33%)	2 (2.74%)	2 (2.74%)
Female (gender)	5 (6.85%)	7 (9.59%)	7 (9.59%)	16 (21.92%)	3 (4.11%)	4 (5.48%)
DA DMet ^1^	10.32 ± 2.21	11.5 ± 2.17	13.11 ± 1.58	14.41 ± 1.46	15.32 ± 0.95	15.73 ± 0.56
DA CEMet ^2^	9.2 ± 2.3	10.25 ± 1.6	11.4 ± 1.4	13.2 ± 1.73	14.6 ± 1.14	15.33 ± 1.03

^1^ Dental age Demirjian method; ^2^ dental age Chronology of Eruption method.

**Table 3:** Difference between CA and DA according to CVM stage.

CVM Stage	Variable	Value	Mean
CVM 1	CA	10 (13.7%)	9.1 ± 1.97
	DA		10.32 ± 2.21
	Δ *		1.22 ± 0.93
CVM 2	CA	12 (16.44%)	10.33 ± 1.5
	DA		11.5 ± 2.17
	Δ *		1.17 ± 0.76
CVM 3	CA	15 (20.55%)	11.67 ± 1.23
	DA		13.11 ± 1.58
	Δ *		1.44 ± 0.517
CVM 4	CA	25 (34.25%)	13.08 ± 1.22
	DA		14.41 ± 1.46
	Δ *		1.33 ± 0.38
CVM 5	CA	5 (6.85%)	14.4 ± 0.89
	DA		15.32 ± 0.95
	Δ *		0.92 ± 0.58
CVM 6	CA	6 (8.22%)	14.83 ± 0.75
	DA		15.73 ± 0.56
	Δ *		0.9 ± 0.43

* Δ = difference between CA and DA.

**Table 4:** Mean CA by CVM depending on gender.

Gender	CVM Stage	CA (Mean)	Gender	CVM Stage	CA (Mean)
Female	1	8.60	Male	1	9.60
Female	2	9.86	Male	2	11.00
Female	3	11.43	Male	3	11.87
Female	4	12.81	Male	4	13.55
Female	5	14.33	Male	5	14.50
Female	6	15.25	Male	6	14.00

## Data Availability

The original contributions presented in this study are included in the article. Further inquiries can be directed to the corresponding author.
